# The Relationship Between Spectral Modulation Detection and Speech Recognition: Adult Versus Pediatric Cochlear Implant Recipients

**DOI:** 10.1177/2331216518771176

**Published:** 2018-05-02

**Authors:** René H. Gifford, Jack H. Noble, Stephen M. Camarata, Linsey W. Sunderhaus, Robert T. Dwyer, Benoit M. Dawant, Mary S. Dietrich, Robert F. Labadie

**Affiliations:** 1 Department of Hearing and Speech Sciences, Vanderbilt University Medical Center, Nashville, TN, USA; 2 Department of Otolaryngology, Vanderbilt University Medical Center, Nashville, TN, USA; 3 Department of Electrical Engineering and Computer Science, Vanderbilt University, Nashville, TN, USA; 4 Department of Biostatistics, Vanderbilt University Medical Center, Nashville, TN, USA

**Keywords:** spectral modulation detection, spectral resolution, cochlear implant, speech recognition, hearing loss

## Abstract

Adult cochlear implant (CI) recipients demonstrate a reliable relationship between spectral modulation detection and speech understanding. Prior studies documenting this relationship have focused on postlingually deafened adult CI recipients—leaving an open question regarding the relationship between spectral resolution and speech understanding for adults and children with prelingual onset of deafness. Here, we report CI performance on the measures of speech recognition and spectral modulation detection for 578 CI recipients including 477 postlingual adults, 65 prelingual adults, and 36 prelingual pediatric CI users. The results demonstrated a significant correlation between spectral modulation detection and various measures of speech understanding for 542 adult CI recipients. For 36 pediatric CI recipients, however, there was no significant correlation between spectral modulation detection and speech understanding in quiet or in noise nor was spectral modulation detection significantly correlated with listener age or age at implantation. These findings suggest that pediatric CI recipients might not depend upon spectral resolution for speech understanding in the same manner as adult CI recipients. It is possible that pediatric CI users are making use of different cues, such as those contained within the temporal envelope, to achieve high levels of speech understanding. Further investigation is warranted to investigate the relationship between spectral and temporal resolution and speech recognition to describe the underlying mechanisms driving peripheral auditory processing in pediatric CI users.

## Introduction

Current cochlear implant (CI) technology yields significant improvement in speech understanding and quality of life for the majority of recipients (e.g., Dowell, Mecklenburg, & Clark, 1986; Horn et al., 1991; Maillet, Tyler, & Jordan, 1995). Speech understanding in noise as well as music perception and appreciation, however, are not only difficult for most CI users but continue to be two primary complaints of CI recipients in the audiology clinic. Reduced spectral resolution contributes to difficulty understanding speech in noise and to poor music perception and appreciation (Jung et al., 2012; Kang et al., 2009; Won, Drennan, Kang, & Rubinstein, 2010) with CI recipients known to have poor spectral resolution (Gifford, Hedley-Williams, & Spahr, 2014; Henry & Turner, 2003; Henry, Turner, & Behrens, 2005; Litvak, Spahr, Saoji, & Fridman, 2007; Saoji & Eddins, 2007; Saoji, Litvak, Spahr, & Eddins, 2009; Won, Drennan, & Rubinstein, 2007). This is generally attributed to a number of factors including (a) a limited number of independent channels despite having up to 22 intracochlear electrodes (Fishman, Shannon, & Slattery, 1997; Friesen, Shannon, Baskent, & Wang, 2001; Friesen, Shannon, & Cruz, 2005), (b) unknown population and location of surviving spiral ganglion cells, and (c) channel interaction due to electric current spread within the cochlea.

For several decades, hearing scientists have been attempting to reduce channel interaction in CI recipients with the goal of improving spectral resolution and spatial selectivity of individual channels in the hopes of achieving improvements in speech understanding in noise and music perception (e.g., Bernstein et al., 2008; Bierer & Litvak, 2016; Bonnet, Frijns, Peeters, & Briaire, 2004; Drennan, Won, Nie, Jameyson, & Rubinstein, 2010; Garadat, Zwolan, & Pfingst, 2013; Koch et al., 2007; Skinner et al., 1994; Smith, Parkinson, & Long, 2013; Srinivasan, Padilla, Shannon, & Landsberger, 2013; Wilson et al., 1991; Won et al., 2012; Zhou, 2017). With the exception of continuous interleaved sampling and n-of-m signal coding using envelope detection and nonsimultaneous stimulation (e.g., Skinner et al., 1994; Wilson et al., 1991), few attempts have yielded more than an incremental change in reducing channel interaction and improving patient outcomes. However, recently introduced computerized tomography image processing techniques now make it possible to estimate the position of implanted CI electrodes relative to the modiolus which contains the primary stimulation targets of intracochlear electrical stimulation, namely the spiral ganglion cells (Labadie et al., 2016; Noble, Gifford, Hedley-Williams, Dawant, & Labadie, 2014; Noble et al., 2016; Noble, Labadie, Gifford, & Dawant, 2013). Further, we are able to use this computerized tomography image-guided analysis to deactivate select intracochlear electrodes predicted—based only on geometric location—to have high probability of channel interaction with neighboring electrodes. To date, we have demonstrated significant improvement in spectral resolution and speech understanding in both quiet and in noise for prelingually (*n* = 26) and postlingually (*n* = 64) deafened adult CI recipients (Labadie et al., 2016; Noble et al., 2013, 2014, 2016) as well as pediatric CI recipients with prelingual onset of deafness (Noble et al., 2016). We have referred to this process as image-guided CI programming.

### Spectral Modulation Detection

In our past studies investigating spectral resolution, we used the quick spectral modulation detection (QSMD) task which is a 5-min task of spectral envelope perception (Gifford et al., 2014). Spectral modulation detection thresholds are generally described as the minimum modulation depth, in dB, required to discriminate a spectrally modulated noise from a flat spectrum noise with the same bandwidth and overall level. There is a reliable, inverse, nonmonotonic relationship between thresholds for spectral modulation detection, in modulation depth, and modulation rate both for adults with normal hearing and with CIs (Saoji & Eddins, 2007; Saoji et al., 2009). Furthermore, research has demonstrated a significant, positive relationship between spectral modulation detection or discrimination and speech understanding for experienced CI users with postlingual onset of deafness (Dorman et al., 2012; Drennan, Anderson, Won, & Rubinstein, 2014; Gifford et al., 2014; Henry & Turner, 2003; Henry et al., 2005; Jung et al., 2012; Saoji et al., 2009; Won et al., 2007; Zhang, Spahr, Dorman, & Saoji, 2013). Of note here is that tasks of spectral ripple discrimination at low ripple densities (<1 ripple per octave) may be more reflective of spectral profile analysis as compared to across-channel spectral resolution (e.g., Anderson et al., 2011; Anderson, Oxenham, Nelson, & Nelson, 2012; Bernstein & Green, 1988). On the other hand, at high ripple densities (>2–4 ripples per octave), CI users’ performance may not be entirely driven by spectral resolution given that current CI electrode configurations and associated frequency assignments are not capable of accurately transmitting more than two to four peaks and valleys per octave. Rather, it is hypothesized that listeners demonstrating ripple discrimination thresholds in this range may be using some combination of spectral and temporal processing.

Most previous studies investigating a relationship between spectral envelope perception and speech understanding have focused on postlingually deafened adult CI recipients—leaving an open question regarding the relationship between CI-mediated spectral resolution and speech understanding for adults and children with prelingual onset of deafness. There are studies reporting poor spectral resolution for pediatric CI recipients—even in the presence of high levels of speech understanding (Jung et al., 2012; Olszewski, Gfeller, Froman, Stordahl, & Tomblin, 2005). In fact, Jung et al. (2012) investigated spectral ripple discrimination and monosyllabic word recognition for 10 pediatric CI users and found no statistically significant correlation; however, this could have been due to both small sample size and the fact that the children’s word recognition scores encompassed a relatively restricted range from 46% to 88% correct (with over half the sample scoring ≥72%). In the same group of listeners, Jung et al. (2012) demonstrated a significant correlation between spectral ripple discrimination and closed-set spondee recognition in steady-state noise. Horn et al. (2017) also demonstrated a significant correlation between the speech reception threshold (SRT) for a closed set of 12 spondees in steady-state noise and spectral ripple discrimination for a group of 15 pediatric CI users. However, they reported that the correlation was significant at two spectral modulation depths (10 and 20 dB) but not across the entire range of depths tested (5–30 dB). In contrast, for their sample of postlingually deafened adult CI users, they reported a significant correlation between spondee-based SRT in steady-state noise and spectral ripple discrimination, across all modulation depths (Horn et al., 2017). Similarly, other studies have also demonstrated significant correlations between spectral modulation detection or discrimination and measures of speech understanding both in quiet and in noise for postlingually deafened adult CI users (Anderson et al., 2012; Drennan et al., 2014; Gifford et al., 2014; Henry & Turner, 2003; Henry et al., 2005; Jeon, Turner, Karsten, Henry, & Gantz, 2015; Litvak et al., 2007; Winn, Won, & Moon, 2016; Won, Moon, Jin, Park, & Woo, 2015) and also hearing aid users (Bernstein et al., 2013; Davies-Venn, Nelson, & Souza, 2015; Shim et al., 2014).

### Motivation for Current Study

There are multiple reports of a significant relationship between speech understanding and spectral modulation detection for postlingually deafened adult CI recipients as well as reports of improved speech recognition following attempts to improve spatial selectivity of intracochlear electrical excitation (e.g., Bierer & Litvak, 2016; Noble et al., 2013, 2014; Zhou, 2016, 2017). In contrast, investigation of our data sets for a group of 18 pediatric (Noble et al., 2016) and 26 prelingually deafened adult CI recipients (Labadie et al., 2016) revealed the following: (a) pediatric CI recipients demonstrated significant improvement on various measures of speech understanding following image-guided CI programming yet did not exhibit improvement for QSMD and (b) pediatric recipients exhibited a smaller range *of QSMD scores* as compared to both postlingually deafened (Noble et al., 2013, 2014) and prelingually deafened adult CI participants (Labadie et al., 2016). These observations motivated the current study aimed at investigating the relationship between spectral modulation detection and speech understanding for a large group of CI recipients—including both children and adults with prelingual onset of deafness.

We have pooled data across various studies to compile a 578-patient sample of adult and pediatric CI recipients for whom we have administered the QSMD as well as tasks of speech understanding in quiet and in noise. We report herein on the relationship between spectral modulation detection, as measured by the QSMD task, and speech recognition in quiet and noise in this group. Our research questions were as follows: (a) Is there a relationship between spectral modulation detection and speech understanding in a large, clinical population of postlingually deafened adult CI users using the QSMD? and (b) is there a relationship between spectral modulation detection and speech understanding for adult and pediatric CI recipients with prelingual onset of deafness?

## Materials and Methods

### Participants

Data were collected for 578 experienced CI recipients. Participants ranged in age from 5.6 to 91.1 years. Table 1 provides summary demographic data for the three groups of participants including mean ages, age at CI, CI brand, as well as mean speech understanding scores. Prelingual onset of deafness was determined on the basis of patient report; however, all prelingually deafened adults reported wearing power hearing aids in early childhood and most exhibited speech production characteristics consistent with prelingual deafness. All pediatric CI recipients had confirmed diagnosis of severe-to-profound sensorineural hearing loss prior to 2 years of age available in the electronic medical record. None of the pediatric CI recipients had any additional disabilities that would have impacted their ability to complete the behavioral tasks such as attention deficit disorder, learning disability, autism, or any additional diagnosis impacting cognitive function.

**Table 1.** Participant Demographics Including Sample Sizes, Age at Implantation, Age at Assessment, Implant Manufacturers, as well as Mean Speech Recognition and QSMD Performance, in Percent Correct.

	Mean age at CI (range)	Mean age at testing (range)	Devices	Mean QSMD, % correct (range)	Mean word rec, % correct (range)	Mean sentences, % correct (range)	Mean sentences at +5 dB% correct (range)
Postlingual adult *n* = 477	62.5 years (19.5–90.5)	65.6 years (20.2–91.0)	AB: 112 Cochlear: 252 MED-EL: 113	61.1% (20–100) *n* = 477	51.8% (0–100) *n* = 477	62.7% (0–100) *n* = 453	29.9% (0–92) *n* = 334
Prelingual adult *n* = 65	42.2 years (18.3–79.8)	46.9 years (19.9–82.1)	AB: 19 Cochlear: 31 MED-EL: 15	47.6% (17–92) *n* = 65	36.2% (0–88) *n* = 65	45.8% (0–100) *n* = 59	18.2% (0–73) *n* = 43
Prelingual pediatric *n* = 36	4.6 years (1.0–10.7)	10.9 years (5.6–17.9)	AB: 11 Cochlear: 24 MED-EL: 1	44.9% (23–87) *n* = 36	61.3% (8–92) *n* = 36	77.0% (10–100) *n* = 36	62.6% (0–97) *n* = 22

*Note*. QSMD = quick spectral modulation detection.

### Stimuli and Listening Conditions

All 578 participants were assessed in the unilateral CI condition. That is, if the patient wore a hearing aid on the nonimplanted ear, that ear was occluded for assessment; or if a patient was a bilateral CI recipient, only the first implanted ear was assessed and reported here. Monosyllabic word recognition was assessed using the Consonant-Nucleus-Consonant (Peterson & Lehiste, 1962) and Lexical Neighborhood Test (Kirk, Pisoni, & Osberger, 1995) for adult and pediatric CI recipients, respectively. Sentence recognition was assessed using the AzBio (Spahr et al., 2012) and Pediatric AzBio (BabyBio; Spahr et al., 2014) sentences for adult and pediatric CI recipients, respectively. Sentence recognition in noise was assessed with colocated speech and noise (S_0_N_0_) in the presence of a multi-talker babble with speech at +5 dB signal-to-noise ratio (SNR). All recorded stimuli were presented from a single loudspeaker placed at 0 degrees at a distance of 1 m from the listener. Speech and QSMD stimuli were presented at a calibrated level of 60 dBA. For individuals with residual acoustic hearing in the implanted or nonimplanted ears, ears were occluded with a foam plug for all sound field assessments. Acoustic hearing thresholds were consistent with moderate sloping to profound sensorineural hearing loss such that given the presentation levels used here, the addition of a foam plug was sufficient. All testing were completed in one of the three laboratory or clinical spaces including either a single-walled, sound-treated booth (laboratory) or a double-walled booth (clinic). All speech and QSMD stimuli were stored on either a DELL Precision 7910 or an HP EliteDesk 800 computer. The output of the PC sound card was routed to a Tannoy Di5 speaker through a GSI 61 audiometer, which served as the amplifier for the stimuli. All stimuli were calibrated in the sound field prior to each assessment using a Larson Davis Soundtrack LxT sound level meter.

The QSMD task employed a three-interval, forced choice procedure based on a modified method of constant stimuli (e.g., Fechner, 1860; Gescheider, 1997). In this task, two of the three intervals contained flat-spectrum noise and the third contained spectral modulation achieved by applying logarithmically spaced, sinusoidal modulation to the broadband carrier (125–5600 Hz). Six trials were presented for each of the five modulation depths (10, 11, 13, 14, and 16 dB) and two modulation rates (0.5 and 1.0 cyc/oct). Each trial was scored as either correct or incorrect, and spectral resolution was described as the overall percent-correct score for the task collapsed across modulation depth and rate with 33% being chance score (for more details, see Gifford et al., 2014). Note that this measure was developed and validated to provide a single description of spectral modulation detection, in percent correct, averaged across modulation depths and rates. That is, we did not assess whether we could reliably extract performance scores for each modulation rate and depth from the QSMD measure. Thus for the purposes of this study, we report on the single measure of spectral modulation detection, in percent correct.

Each pediatric participant was given training to provide familiarization with the task as well as the scoring method to ensure that the children were able to accurately complete the task. A touch-screen monitor was located in the booth to the side of the participant positioned according to the participant’s handedness. The participant recorded his or her response by touching a box on the screen labeled 1, 2, or 3 to indicate which interval she or he believed was different from the others. No feedback was provided on any of the experimental trials, though feedback was provided for the training session. The training session consisted of a series of practice trials and was generally 3 min for the children and 1 min for the adults.

## Results

Table 1 displays mean speech recognition and QSMD scores, in percent correct, for all three groups. Statistical analyses were completed comparing speech recognition and spectral resolution across groups. Nonparametric statistical analysis was completed using an independent samples Kruskal–Wallis analysis of variance as the data were not normally distributed. In an attempt to minimize the contribution of floor and ceiling effects, we converted all speech recognition scores from percent correct to rationalized arcsine units or RAU (Studebaker, 1985) prior to analysis. There was a significant effect of group for QSMD (H_2_ = 46.4, *p* < .001, η^2 ^= 0.14), monosyllabic word recognition (H_2_ = 29.3, *p* < .001, .10), sentence recognition (H_2_ = 27.7, *p* < .001, η^2 ^= 0.10), and sentence recognition in noise (H_2_ = 32.4, *p* < .001, η^2 ^= 0.10). Post hoc analysis was completed using O. J. Dunn’s (1964) test of multiple comparisons. For all measures of speech recognition, all three participant groups were significantly different from one another. For monosyllabic word recognition, pediatric CI users scored significantly higher than prelingual adults (Q = 5.1, *p* < .05) and postlingual adults (Q = 2.8, *p* < .05), and postlingual adults scored significantly higher than prelingual adults (Q = 4.4, *p* < .05). For sentence recognition in quiet, pediatric CI users scored significantly higher than prelingual adults (Q = 5.2, *p* < .05) and postlingual adults (Q = 3.4, *p* < .05), and postlingual adults scored significantly higher than prelingual adults (Q = 3.7, *p* < .05). For sentences at + 5 dB SNR, pediatric CI users scored significantly higher than prelingual adults (Q = 5.7, *p* < .05) and postlingual adults (Q = 4.4, *p* < .05), and postlingual adults scored significantly higher than prelingual adults (Q = 3.3, *p* < .05). For QSMD, postlingual CI users scored significantly higher than prelingual adults (Q = 5.2, *p* < .05) and pediatric CI users (Q = 4.9, *p* < .05); however, prelingual adults and pediatric CI users’ QSMD scores were not significantly different (Q = 0.8, *p* > .05).

**Figure 1.**
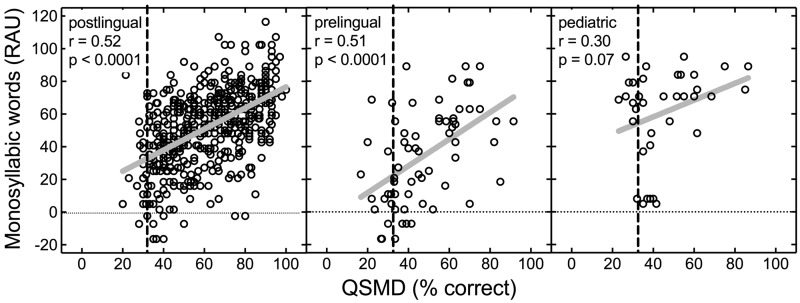
Individual data for monosyllabic word recognition as a function of spectral modulation detection using the QSMD test, both in percent correct. The vertical dashed line represents chance performance on the QSMD task. Sample sizes for the postlingual adults, prelingual adults, and prelingual pediatric CI recipients are 477, 65, and 36, respectively. Solid gray lines represent the linear regression function for each panel. Pearson’s correlation coefficients and associated *p* values are displayed in each panel. QSMD = quick spectral modulation detection; RAU = rationalized arcsine units.

Figure 1 displays monosyllabic word recognition as a function of QSMD for the 477 postlingual adults, 65 prelingual adults, and 36 pediatric CI recipients. Pearson’s correlation analysis was completed for each of the three subject groups. Significant correlations were found for both adult groups (postlingual: *r* = .52, *n* = 477, *p* < .0001; prelingual: *r* = .51, *n* = 65, *p* < .0001). For the pediatric CI recipients, the correlation between monosyllabic word recognition and QSMD was not statistically significant (*r* = .30, *n* = 36, *p* = .07).

**Figure 2.**
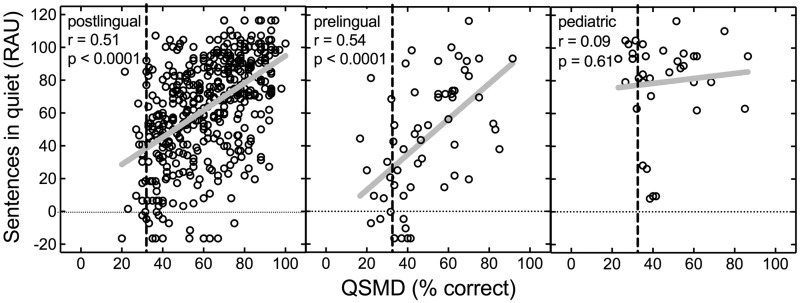
Individual data for sentence recognition, in quiet, as a function of spectral modulation detection using the QSMD test, both in percent correct. The vertical dashed line represents chance performance on the QSMD task. Sample sizes for the postlingual adults, prelingual adults, and prelingual pediatric CI recipients are 456, 59, and 36, respectively. Solid gray lines represent the linear regression function for each panel. Pearson’s correlation coefficients and associated *p* values are displayed in each panel. QSMD = quick spectral modulation detection; RAU = rationalized arcsine units.

Figure 2 displays sentence recognition in quiet as a function of QSMD for the different groups. Similar to monosyllabic word recognition, significant correlations between sentence recognition in quiet and QSMD were found for both adult groups (postlingual: *r* = .51, *n* = 456, *p* < .0001; prelingual: *r* = .54, *n* = 59, *p* < .0001). For the pediatric CI recipients, the correlation between QSMD and sentence scores was not statistically significant, and because of the low magnitude of the relationship (*r* = .09, *n* = 36, *p* = .61), this was unlikely to have arisen from limited power.

**Figure 3.**
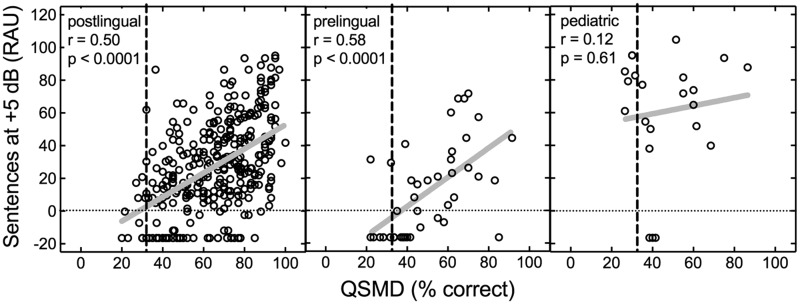
Individual data for sentence recognition in noise (+5 dB SNR) as a function of spectral modulation detection using the QSMD test, both in percent correct. The vertical dashed line represents chance performance on the QSMD task. Sample sizes for the postlingual adults, prelingual adults, and prelingual pediatric CI recipients are 334, 43, and 22, respectively. Solid gray lines represent the linear regression function for each panel. Pearson’s correlation coefficients and associated *p* values are displayed in each panel. QSMD = quick spectral modulation detection; RAU = rationalized arcsine units.

Figure 3 displays sentence recognition at + 5 dB SNR as a function of QSMD for all participant groups. Significant correlations between sentence recognition at +5 dB SNR and QSMD were found for both adult groups (postlingual: *r* = .50, *n* = 334, *p* < .0001; prelingual: *r* = .58, *n* = 43, *p* < .0001. For the pediatric CI recipients, the correlation between sentence recognition at +5 dB SNR and QSMD was not statistically significant (*r* = .12, *n* = 22, *p* = .61).

To investigate whether the strength of the relationship between measures of speech recognition and QSMD was different across the subject groups, we completed a global test of the interaction effects for group with QSMD and speech recognition performance using a generalized linear model. Generalized linear modeling revealed no statistically significant interaction effect of group with QSMD for either monosyllabic word recognition (Wald χ^2^[*df* = 2] = 1.72, *p* = .422) or sentence recognition at +5 dB SNR (Wald χ^2^[*df* = 2] = 2.99, *p* = .224). There was, however, a statistically significant interaction effect of group with QSMD for sentences in quiet (Wald χ^2^[*df* = 2] = 7.69, *p* = .021). Post hoc pairwise comparisons of the differences among the slopes revealed that the associations between QSMD and sentence recognition in quiet were significantly greater for the two adult groups than that observed in the pediatric group (postlingual adult vs. pediatric: *z* = 2.63, *p* = .009; postlingual adult vs. pediatric: *z* = 2.34, *p* = .019).

To investigate the possibility that QSMD may have been influenced by age at testing for the pediatric group, we completed Pearson’s correlation analyses between QSMD and age at testing. The correlation between listener age and QSMD for the pediatric participants was not significant (*r* = .31, *n* = 36, *p* = .06). We also ran correlation analyses for QSMD score and listener age for the adult listeners. We found a significant negative correlation between QSMD and listener age for the postlingually deafened adults (*r* = −.11, *n* = 477, *p* = .01), similar to that reported by Landsberger, Padilla, Martinez, and Eisenberg (2018), but not for the prelingually deafened adults (*r* = .02, *n* = 65, *p* = .86). The negative correlation between QSMD and listener age suggests that older CI recipients with postlingual onset of deafness have poorer spectral resolution than younger CI recipients. However, we observed chance performance (±10%) for 21 of the 36 pediatric participants providing evidence for floor effects in this sample. Although the effect size was small for this correlation (*r* = −.11), this finding corroborates results presented by Landsberger et al. (2018) and thus warrants further investigation. Should we determine that older CI recipients have poorer spectral resolution, this could influence patient counseling, prediction of CI outcomes, and ultimately determine optimal signal processing parameters for older CI recipients.

## Discussion

The current data set demonstrates a significant correlation between all measures of speech understanding and QSMD for both prelingually and postlingually deafened adult CI recipients; however, there was no statistically significant correlation between speech understanding and QSMD for prelingually deafened pediatric CI recipients. In fact, despite obtaining high levels of speech understanding, pediatric CI recipients generally exhibited poor QSMD scores, which rarely exceeded 60% correct.

### Spectral Resolution and Speech Understanding for Pediatric CI Users

There are numerous reports showing a significant relationship between monosyllabic word recognition and spectral modulation discrimination/detection (Anderson et al., 2012; Drennan et al., 2014; Gifford et al., 2014; Jeon et al., 2015; Jung et al., 2012; Litvak et al., 2007; Saoji & Eddins, 2007; Saoji et al., 2009; Won et al., 2007), speech recognition in noise and spectral modulation detection (Dorman et al., 2012; Horn et al., 2017; Jung et al., 2012; Zhang et al., 2013), as well as consonant and vowel recognition and spectral modulation discrimination (Henry & Turner, 2003; Henry et al., 2005). However, most studies investigating the relationship between spectral envelope perception and speech understanding have focused on postlingually deafened adults with CIs.

There are reports of pediatric CI recipients exhibiting poor spectral resolution yet high levels of speech understanding. A number of studies have investigated tasks of spectral resolution, speech understanding, and speech production within the context of tonal languages for pediatric CI recipients—the latter of which is highly dependent upon spectral resolution as temporal envelope cues limit pitch perception to frequencies below approximately 300 Hz (e.g., Burns & Viemeister, 1976, 1981). The general findings have been that pediatric CI recipients who demonstrate poor tone perception, discrimination, and speech production are still able to achieve high levels of speech understanding including monosyllabic and disyllabic word recognition (Lee, van Hasselt, Chiu, & Cheung, 2002; Peng, Tomblin, Cheung, Lin, & Wang, 2004; Yuan et al., 2009). Further, Hsiao (2008) demonstrated poor melodic pitch recognition yet nearly perfect lyric (i.e., words sung in music) recognition for a group of 20 pediatric CI recipients with prelingual onset of deafness—all of whom were native Mandarin speakers.

Olszewski et al. (2005) examined familiar melody recognition for 40 pediatric CI users and found no correlation between melody recognition and speech understanding using tasks of monosyllabic word recognition and sentence recognition in quiet. In fact, Olszewski et al. (2005) stratified their pediatric CI group into those with pre- and postlingual onset of deafness and demonstrated no correlation between melody recognition and speech recognition for either group. They did, however, find a significant correlation between melody (task of spectral resolution) and sentence recognition for a group of 57 adult CI recipients—similar to the results in the current study.

Jung et al. (2012) obtained estimates of spectral envelope discrimination as well as pitch discrimination for 10 pediatric CI users (mean age of 12.1 years). They reported no correlation between monosyllabic word recognition and spectral ripple discrimination—a finding similar to the current study—nor between monosyllabic word recognition and pitch discrimination—a finding similar to that reported by Hsiao (2008). They did, however, report a significant correlation between SRTs for a closed set of 12 spondee words in steady-state noise and spectral ripple discrimination (Jung et al., 2012). Using similar tasks, Horn et al. (2017) also demonstrated a significant correlation between spondee-based SRTs in steady-state noise and spectral ripple discrimination for a group of 15 pediatric CI users. However, this relationship was not observed across the entire range of modulation depths tested (5–30 dB) as seen for the adult population. It is quite possible that SRTs for a closed set of spondees are more influenced by top-down processing abilities than by peripheral sensory function. The reason is that spondaic words have significantly greater contextual influence than monosyllabic and disyllabic words (e.g., Moulin & Richard, 2015). Context influence is dependent upon a number of listener and linguistic factors. Listener-specific factors known to impact contextual influence include age, education, cognition, and degree of hearing loss (e.g., Benichov, Cox, Tun, & Wingfield, 2012). Linguistic factors impacting contextual influence include word occurrence frequency (both in written and spoken contexts), number of items in the test, repetition, and phonological neighborhood density (e.g., Brysbaert & New, 2009; Miller, Heise, & Lichten, 1951; Moulin & Richard, 2015; Nittrouer & Boothroyd, 1990). Thus, the theory is that a task of peripheral spectral resolution would have a greater relationship with a linguistic task more heavily dependent on bottom-up processing (e.g., monosyllabic word recognition) as compared to tasks where lexical context is more dominant (e.g., sentence recognition). If we are to fully understand the relationship between underlying spectral resolution and speech understanding, it is necessary to assess various estimates of speech understanding, including tasks reliant on bottom-up processing (i.e., monosyllabic word recognition) as well as top-down processing (i.e., closed-set tasks and high-context sentence recognition) such as the current study.

### Pediatric CI Recipients: Different Underlying Mechanism Driving Performance

The present findings, along with those reported elsewhere (Jung et al., 2012; Olszewski et al., 2005), provide support for the supposition that congenitally deafened pediatric CI recipients may not depend upon spectral resolution for speech recognition in the same manner as adult CI recipients. Prelingually deafened adults demonstrated a correlation between QSMD and speech understanding, suggesting that despite an extended period of auditory deprivation, these prelingually deafened adults developed the ability to utilize spectral cues. Important to note here, however, is the fact that all prelingually deafened adult listeners in the current study had worn hearing aids prior to implantation and had communicated primarily via listening and spoken language.

It is possible that pediatric CI recipients are making use of different cues, such as those contained within the temporal envelope, which have been shown to yield high levels of consonant recognition in normal hearing adults (e.g., Rosen, 1992; van Tasell, Soli, Kirby, & Widin, 1987). Further investigation is warranted to investigate the relationship between spectral resolution, speech recognition, and underlying mechanisms driving speech understanding—particularly peripheral-based measures involving bottom-up processing—in pediatric CI users. We plan to continue following this pediatric cohort to determine when and if they will mirror the trends exhibited by our group of prelingually deafened adult CI recipients.

While the relationship between spectral resolution and speech understanding for pediatric CI users is unclear, multiple researchers have demonstrated that improvements in electrode spatial selectivity yield improvements in speech recognition in quiet and noise for postlingually deafened adult CI users (e.g., Bierer & Litvak, 2016; Labadie et al., 2016; Noble et al., 2013, 2014; Zhou, 2016, 2017; but see Berenstein, Mens, Mulder, & Vanpoucke, 2008). Furthermore, in the current report, we demonstrated significant correlations between all measures of speech understanding and spectral modulation detection for adult CI users—both with prelingual and postlingual onset of deafness. At a fundamental level, word recognition is dependent upon spectral resolution of the individual components and formant transitions; however, though tasks of monosyllabic word recognition do not have the rich lexical content available in sentence recognition tasks, this is still a linguistic task offering the listener cues based on phonotactic probability (e.g., Vitevitch, Luce, Charles-Luce, & Kemmerer, 1997; Vitevitch, Luce, Pisoni, & Auer, 1999). Thus both measures should reflect sensory function for the CI recipient. This point is an important consideration as various attempts are made to improve channel interaction for intracochlear electrical stimulation.

### Limitations

Could the lack of correlation between speech understanding and spectral modulation detection in the pediatric CI population be due to task difficulty or lack of neural maturation? Regarding QSMD task difficulty, we completed a practice session for each participant in which children were asked to identify the “different” sound out of three possibilities. All children demonstrated a thorough understanding of the task and were generally able to identify the signal with the modulated spectrum for trials with the largest modulation depths, despite the fact that 21 of 36 pediatric participants performed at or within 10 percentage points of chance for the QSMD measure. In addition, an experimenter sat in the booth with the younger children to ensure that they remained on task during experimentation. Furthermore, there was no significant correlation between QSMD score and listener age for the pediatric CI recipients. This is an important point because it suggests that spectral modulation detection either may not be related to the listener age for prelingually deafened, pediatric CI recipients, or the age range over which these participants did not capture the maturation effects for spectral resolution. Of course, it is still possible that the QSMD task may require modification to be more pediatric friendly for future investigation, but we are confident that the *task itself* was not a limiting factor.

Regarding neural maturation, Sheffield, Simha, Jahn, and Gifford (2016) administered the QSMD test to 19 children with normal hearing (mean age = 9.3 years) using both unprocessed stimuli and CI simulations. Even with the unprocessed stimuli, the normal hearing children did not achieve ceiling performance with scores ranging from below chance (33%) to 90% correct. Interestingly, the QSMD scores were significantly correlated with listener age for this group of normal hearing children suggesting that either higher level spectral resolution had not yet reached maturity for the children in that study (6–12 years) or the task was too difficult for the youngest participants. Given that the children ranged in age from 5 to 17 years in the current study, a possible explanation for the lack of a correlation is that spectral resolution had not yet reached maturity in our pediatric population. Indeed, there are a number of previous studies documenting that while peripheral spectral resolution is mature by 3 to 6 months of age (e.g., Abdala & Folsom, 1995; Lau & Werner, 2012; Montgomery & Clarkson, 1997; Spetner & Olsho, 1990), even children with normal hearing demonstrate poorer than normal performance on tasks of spectral resolution until adolescence (e.g., Hall & Grose, 1991; Moore, Cowan, Riley, Edmondson-Jones, & Ferguson, 2011; Sheffield et al., 2016; Werner, 1996). Researchers have also demonstrated that poorer spectral resolution in children is most likely due to nonsensory factors, such as poorer processing efficiency (e.g., Hall & Grose, 1991; Moore et al., 2011). Poor processing efficiency can manifest as higher masked thresholds (Allen et al., 1989; Hall & Grose, 1991; Irwin et al., 1986) and greater intra- and intersubject variability (Allen et al., 1989; Moore et al., 2011) on various behavioral tasks. Therefore, although we found no statistically significant correlation between QSMD and listener age (*r* = .31, *n* = 36, *p* = .06), it is possible that further investigation with larger sample sizes and broader range of ages may prove otherwise.

Landsberger et al. (2018) investigated spectral-temporal modulation detection for a group of 20 pediatric CI recipients ranging in age from 5 to 13 years as well as a control group of 20 children with normal hearing over the same age range. They found no relationship between listener age and spectral resolution for the pediatric CI recipients; however, there was a significant correlation between listener age and spectral resolution for the control group (Landsberger et al., 2018)—also consistent with the findings reported by Sheffield et al. (2016). In contrast, however, Kirby, Browning, Brennan, Spratford, and McCreery (2015) demonstrated a significant correlation between listener age and spectral resolution for a group of 15 children aged 6 to 16 years with mild to moderate to severe sensorineural hearing loss. Thus, they demonstrated that children with less severe hearing losses exhibited age-related maturation in spectral resolution as observed in children with normal hearing. Pediatric CI recipients, on the other hand, exhibit differential maturation of central auditory function—specifically with respect to spectral resolution—including a longer or shallower trajectory. This may help explain the current findings that pediatric CI recipients are achieving high levels of speech understanding—significantly higher than even our large group of postlingually deafened adult CI recipients—despite exhibiting poor spectral resolution and that spectral resolution does not appear to be significantly correlated with listener age.

If children with prelingual onset of deafness are able to achieve high levels of auditory only speech understanding despite significantly poorer spectral resolution than exhibited by adult CI recipients, what mechanism(s) are driving speech understanding? There is evidence from the literature examining speech recognition in noise for children with normal hearing. Children with normal hearing require higher SNRs for adult-like recognition of speech (e.g., Baker et al., 2014; Buss et al., 2016; Buss, Leibold, Porter, & Grose, 2017; Corbin et al., 2016; Elliot, 1979; Holder et al., 2016; McCreery et al., 2010; Stuart, 2005) and require broader audibility bandwidths than adults to achieve asymptotic speech understanding (e.g., McCreery & Stelmachowicz, 2011; Mlot et al., 2010; Stelmachowicz et al., 2001). As mentioned previously, researchers have implicated poorer processing efficiency for children who generally require higher SNR than adults for comparable detection (e.g., Hall & Grose, 1991). The exact underlying mechanism for processing efficiency is not known, though it is believed to involve central processing and could involve various cognitive processes including working memory, attention, and effort. There is evidence that both vocabulary (Klein, Walker, Kirby, & McCreery, 2017; McCreery et al., 2017) and working memory (McCreery et al., 2017) significantly impact speech recognition in noise for children. In the current study, we had obtained the estimates of receptive vocabulary (Peabody Picture Vocabulary Test, 4th edition, PPVT-4; L. M. Dunn & Dunn, 2007) and nonverbal intelligence (Leiter International Performance Scale, 3rd edition; Roid, Miller, Pomplun, & Koch, 2013) for 16 of the 36 children with CI. The mean standard scores were 82.8 (range: 63–130) and 108.6 (range: 92–128) for PPVT and Leiter, respectively. For these 16 children for whom we had obtained PPVT scores, we ran Pearson’s correlation analyses and found a significant correlation between PPVT and sentence recognition in noise (*r* = .61, *n* = 16, *p* = .025) but no significant correlation between PPVT and monosyllabic words (*r* = .40, *n* = 16, *p* = .13) or sentences in quiet (*r* = .47, *n* = 16, *p* = .06). Thus, these results are generally consistent with McCreery et al. (2017) who found a significant relationship between PPVT scores and sentence recognition but not with isolated words.

The choice of materials could have also potentially influenced the outcomes. Both groups of adult participants were assessed with the same measures of speech recognition. The pediatric CI recipients, however, were administered tests that were developmentally appropriate for the age range as outlined by the pediatric minimum speech test battery (Uhler, Warner-Czyz, & Gifford, 2017). Using these developmentally appropriate measures, over half of the pediatric CI population scored above 80% correct for sentences in quiet. Thus, it is possible that ceiling effects could have influenced the results. On the other hand, ceiling effects were not an issue for either monosyllabic words or sentences at +5 dB SNR. While it is not likely that the choice of materials influenced the outcomes, we cannot definitively state otherwise at this time.

Finally, it is important to acknowledge that our pediatric CI sample (*n* = 36) was much smaller than our populations of postlingually deafened (*n* = 477) and prelingually deafened (*n* = 65) adult CI recipients. Thus, we cannot rule out the fact that small sample size may have contributed to the differential findings across the groups. Recruitment of pediatric CI recipients for behavioral research participation is ongoing, and we hope to further investigate the relationship between spectral resolution and speech understanding.

### Clinical Implications and Directions for Future Research

Understanding the underlying mechanisms driving speech understanding abilities in pediatric CI recipients is not only necessary for theoretical purposes, but this information is critical to maximize a child’s auditory abilities in the context of CI programming and current signal coding strategies. Clinicians have access to a variety of signal coding strategies all focusing on different aspects of the incoming stimulus. For example, there are current-steering strategies designed to provide greater spectral representation of incoming stimuli (e.g., Fidelity-120, Optima), there are strategies aimed at providing temporal fine structure in the apical channels via variable rate stimulation (e.g., fine structure processing, fine structure 4), and there are higher rate strategies specifically designed to provide fine detail for temporal envelope representation at each stimulated electrode (e.g., HiRes, high-rate Advanced Combination Encoder, and high definition continuous interleaved sampling). Up to this point, most clinicians have approached pediatric CI programming with the thought that what has been good for adult recipients is also good for pediatric recipients. While it is important to note here that neither current-steering strategies nor fine structure processing strategies are FDA approved for use with children, many pediatric audiologists are using these strategies and reporting their findings with their pediatric population (e.g., Chang, Yang, Lin, Liu, & Wu, 2009; Han et al., 2009; Lorens, Zgoda, Obrycka, & Skarzynski, 2010a; Lorens, Zgoda, & Skarzynski, 2010b; Melo, Bevilacqua, Costa, & Moret, 2013; Riss et al., 2011). Should we determine that pediatric CI users are more reliant on temporal coding for speech understanding, we may need to adapt our clinical philosophies to provide greater representation of temporal envelope and also possibly temporal fine structure. Clearly, much research is needed before such recommendations are made.

## Summary and Conclusion

Adult CI recipients have relatively poor spectral resolution, yet demonstrate a significant correlation between spectral envelope perception and speech understanding. In the current study, we have replicated this finding between performance on the QSMD task and various measures of speech understanding for 542 adult CI recipients (477 postlingual and 65 prelingual). A group of 36 pediatric CI recipients, however, did not demonstrate a relationship between spectral envelope perception and speech understanding in quiet or in noise. Our findings along with various others referenced herein provide support for the possibility that pediatric CI recipients with prelingual onset of deafness may not depend upon spectral resolution for speech understanding in the same manner as adult CI recipients. It is possible that prelingually deafened pediatric CI users are making use of different cues than adult CI users, such as those contained within the temporal envelope, to achieve high levels of speech understanding. Further investigation is warranted to investigate the relationship between spectral and temporal resolution, speech understanding, and underlying mechanisms driving bottom-up processing in both pediatric and adult CI users with prelingual deafness. Clinical implications regarding signal processing strategies and recommended aural habilitation may depend upon the known underlying mechanisms driving performance.

## Authors’ Note

The methods of this study were approved by the Vanderbilt Institutional Review Board (IRB numbers 130229, 101509, and 090155). These data were presented at the 2017 Conference on Implantable Auditory Prostheses in Tahoe City, CA held July 17–21, 2017.
